# What factors are associated with the poor prognosis of anal adenocarcinoma compared with low-lying rectal adenocarcinoma based on a population analysis: A propensity score matching study

**DOI:** 10.1371/journal.pone.0219937

**Published:** 2019-07-30

**Authors:** Qinghua Wang, Jianfei Fu, Xiaoxiao Chen, Cheng Cai, Hang Ruan, Jinlin Du

**Affiliations:** Department of Colorectal and Anal Surgery, Jinhua Central Hospital, Jinhua, Zhejiang Province, China; University of Texas MD Anderson Cancer Center, UNITED STATES

## Abstract

**Purpose:**

Anal adenocarcinoma (AA) represents a rare condition, and little is known about the predictive factors of the outcomes or the optimal TNM staging system for curable AA. Using population-based data, we preliminarily sought to determine the prognostic factors and evaluate the existing T and N staging criteria of AA.

**Methods:**

We analyzed the Surveillance, Epidemiology, and End Results 18 database to identify patients 20–80 years old who were diagnosed with AA or rectal adenocarcinoma (RA) and underwent abdominal perineal resection between 2004 and 2012. The difference between Kaplan-Meier survival curves was estimated by a log-rank test. A Cox proportional hazard regression model was used to adjust the effects of other covariates on survival in the propensity score-matched cohort, including age, gender, race, marital status, histology, grade of differentiation, tumor size, number of positive lymph nodes, radiotherapy, and chemotherapy.

**Results:**

Compared to patients with RA, patients with AA had a worse CSS after controlling for other covariates (hazard ratio [HR], 1.96; 95% confidence interval [CI], 1.25–3.07; P<0.01). For AA, the increasing tumor size (2–5 cm: HR, 0.62; 95% CI, 0.29–1.32; P>0.05; >5 cm: HR, 1.01; 95% CI, 0.49–2.07; P>0.05) had no significant influence on survival. The number of positive lymph nodes (1–3: HR, 2.93; 95% CI, 1.55–5.53; P<0.01; ≥4: HR, 4.24; 95% CI, 2.08–8.62; P<0.01) significantly influenced survival.

**Conclusions:**

AA confers a worse prognosis than RA does. The T staging criteria of anal carcinoma, dominated by tumor size, seem to be invalid for AA, while the number of positive lymph nodes is a prognostic factor.

## Introduction

Anal adenocarcinoma (AA), which is thought to originate from the columnar epithelium lining the anal glands, is a rare condition, accounting for approximately 1.5%-2.5% of all digestive system cancers[1, 2]. However, the worldwide incidence rate has steadily increased over the years[2]. The anatomical terminology and pathogenesis of this disease are controversial, leading to disparities in both diagnosis and treatment[3]. As described in detail previously[3], clinical differences have also been noted for AA, including a more advanced presentation and malignancy, and a worse prognosis compared with epidermoid cancer, which encompasses the majority of anal cancers, in addition to the histological differences. To the best of our knowledge, the predictive factors related to the poor prognosis of AA are not clear, and there are few published reports to date describing the topic. Furthermore, despite the recommendation that the management of AA is referred to as that of rectal adenocarcinoma (RA), the TNM staging system defers from either RA or anal epidermoid cancer due to the anatomical distinction and lymphatic drainage pathway. Nevertheless, AA is closest to low-lying RA anatomically and histologically, and the two have a very similar surgical approach. Therefore, an actuarial comparison was conducted to illustrate the similarities and differences between AA and low-lying RA.

We sought to better determine the factors relative to poor prognosis and to explore whether the existing T and N staging criteria of anal carcinoma are suitable for AA or not in a larger cohort of patients from the Surveillance, Epidemiology, and End Results (SEER) database.

## Methods

### Study population

As described in detail previously[4, 5], the Surveillance, Epidemiology, and End Results (SEER) program is an authoritative American Cancer Information Database that is sponsored by the National Cancer Institution with the aim of collecting information about cancer incidence and survival. The current SEER database collects and publishes cancer data from 18 population-based cancer registries among 14 states across the United States, representing approximately 28% of the United States population. The SEER database lacks identifying information, and it is publicly available for cancer epidemiology and health policy studies (http://seer.cancer.gov/). The SEER database is collected and released annually, reflecting the most updated information. We obtained permission to access the research data (Reference Number: 10263-Nov2015). The study was approved by the review board of Zhejiang University Jinhua hospital. SEER Stat software (SEER Stat 8.1.2) was utilized to identify patients diagnosed with histologically confirmed adenocarcinoma of the anal canal or low rectum without metastatic lesions from 2004–2012. The patients diagnosed after 2012 were excluded to ensure an adequate follow-up duration. The year and age at diagnosis, gender, race, marital status, histological type, differentiated grade, radiotherapy, chemotherapy, survival time and cause of death were retrieved from the SEER database.

**Fig 1:**
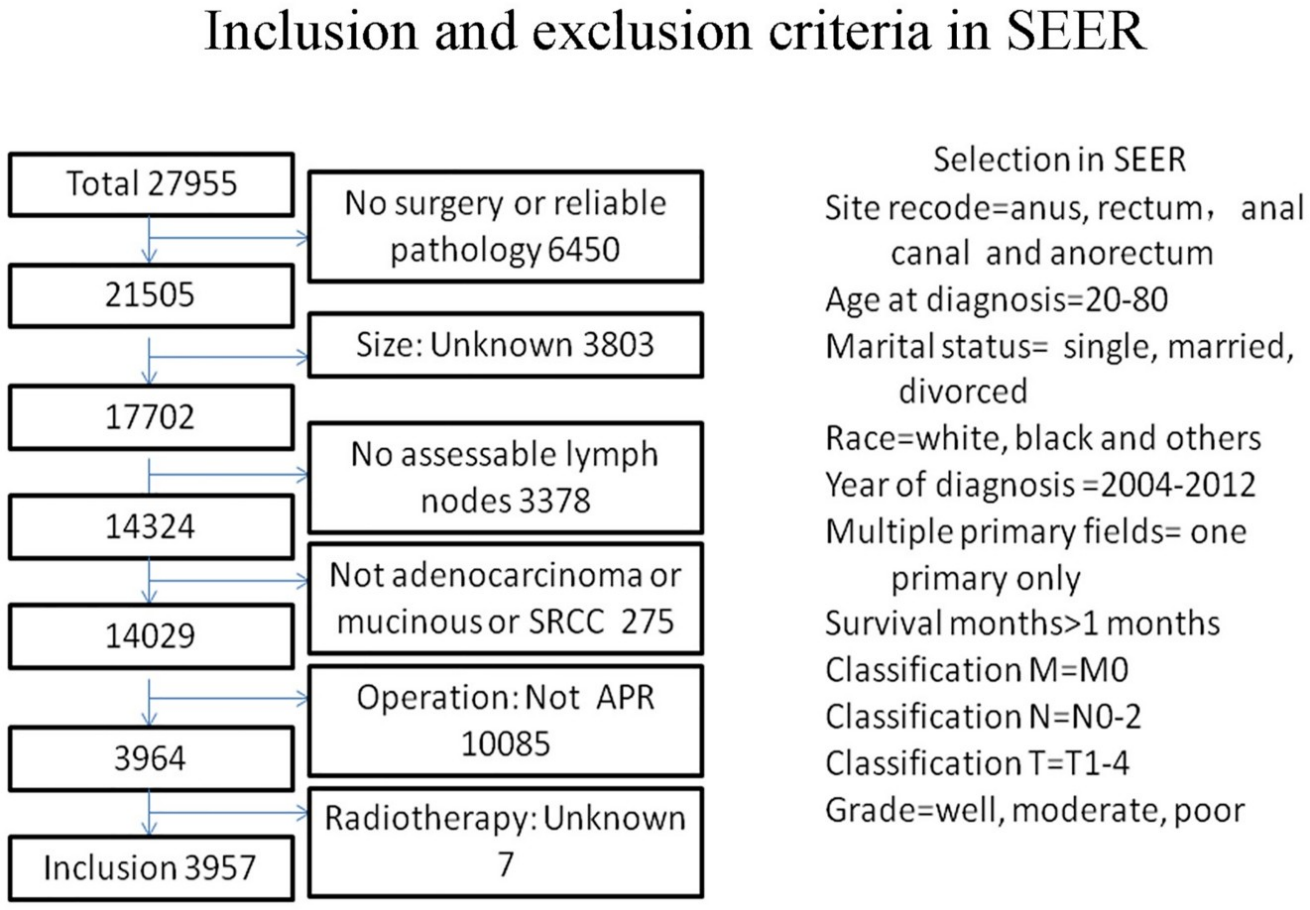
The inclusion and exclusion criteria in seer.

The specific inclusion criteria were as follows: (1) site record ICD-O-3 was limited to anus, anal canal, anorectum and rectum (C20.9, C21.0-C21.2, C21.8); (2) histological type ICD-O-3 was limited to 8140/3, 8210/3, 8215/3, 8255/3, 8261/3, 8263/3, 8480/3, 8481/3, 8490/3, 8574/3; (3) patients without distant metastases; and (4) patients who underwent abdominal perineal resection (APR). The exclusion criteria were as follows: (1) patients without documentation of race or age at diagnosis; patients younger than 20 years or older than 80 years; (2) patients with multiple primary tumors; (3) patients who survived less than one month (Fig 1).

### Variable declaration

Race was divided into white, black and others. Marital status was grouped as married, single or divorced. Histology was grouped as adenocarcinoma, mucinous adenocarcinoma or signet ring cell carcinoma (SRCC). Differentiated grades were divided into well, moderately, and poorly differentiated. Tumor size was grouped as ≤2 cm, 2–5 cm or>5 cm. N-classification was divided into N0 (0 positive modes), N1 (1–3 positive nodes), and N2 (≥4 positive nodes). Radiotherapy was divided into yes or no, regardless of the sequence. Chemotherapy was divided into yes or no, regardless of the sequence.

### Statistical analyses

Survival curves were calculated using the Kaplan-Meier method. Differences in survival curves between the various cancer types were computed using a log-rank test. The cancer-specific survival (CSS) was calculated from the date of diagnosis to the date of cancer death. Death attributed to other causes was defined as a censored observation. Multivariate analysis was conducted using Cox regression analysis to identify the independent effect of cancer type on survival controlling for age, gender, race, marital status, histology, grade of differentiation, tumor size, number of positive lymph nodes, radiotherapy, and chemotherapy. A separate univariate regression analysis was conducted to identify the independent predictors of survival among patients with AA in which age, gender, race, marital status, histology, grade of differentiation, tumor size, number of positive lymph nodes, radiotherapy, and chemotherapy were included as covariates. The method of propensity score matching (PSM) was used to balance differences in the baseline characteristics between the RA and AA groups. The propensity score was calculated by logistic regression including covariates of age, gender, race, marital status, histology, grade of differentiation, tumor size, number of positive lymph nodes, radiotherapy, and chemotherapy. The adjusted cohort was used to validate the impact of independent predictors on outcome. When the two-sided P-value was less than 0.05, the difference was considered statistically significant. R3.3.2 software (http://www.r-project.org/) and STATA/SE 12.0 software (StataCorp LP, College Station, TX, USA) were used to perform the statistical analyses.

## Results

The cut-off date for follow-up was November 2012, and there was a median follow-up of 44.0 months (range 1–119 months). A total of 3,957 eligible patients were included in the analysis. The 5-year CSS survival rate was 73.95%. The median age was 60 years (IQR 51–69 years old). Of the 3,957 patients in the study, 3821 (96.56%) were RA patients, and 136 (3.44%) were AA patients. The detailed clinicopathologic characteristics between the two groups are presented in Table 1.

**Table 1:** Descriptive characteristics of 3957 patients of study population within the Surveillance, Epidemiology, and End Results (SEER) Medicare-linked database and 272 propensity score-matched patients.

Characteristics	Entire cohort (n = 3957)			Propensity score-matched cohort (n = 272)		
	RA, n(%)	AA, n(%)	*P* Value	RA, n(%)	AA, n(%)	*P* Value
	n = 3821 (96.56)	N = 136 (3.44)		136 (50)	136 (50)	
**Age**			*0*.*311*			*0*.*754*
<50	872(22.82)	26(19.12)		24(17.65)	26(19.12)	
≥50	2949(77.18)	110(80.88)		112(82.35)	110(80.88)	
**Gender**			*0*.*935*			*0*.*801*
Female	1446(37.84)	51(37.50)		49(36.03)	51(37.5)	
Male	2375(62.16)	85(62.50)		87(63.97)	85(62.5)	
**Race**			*0*.*097*			*0*.*826*
White	3092(80.92)	102(75.00)		103(75.74)	102(75)	
Black	355(9.29)	20(14.71)		17(12.5)	20(14.71)	
Other	374(9.79)	14(10.29)		16(11.76)	14(10.29)	
**Marital status**			*0*.*057*			*0*.*536*
Married	2421(63.36)	74(54.41)		75(55.15)	74(54.41)	
Single	586(15.34)	22(16.18)		16(11.76)	22(16.18)	
Divorce	814(21.30)	40(29.41)		45(33.09)	40(29.41)	
**Histology**			*0*.*000*			*0*.*710*
Adenocarcinoma	3475(90.94)	108(79.41)		110(80.88)	108(79.41)	
Mucinous	303(7.93)	24(17.65)		24(17.65)	24(17.65)	
SRCC	43(1.13)	4(2.94)		2(1.47)	4(2.94)	
**Grade**			*0*.*001*			*0*.*726*
Well	271(7.09)	18(13.24)		21(15.44)	18(13.24)	
Moderate	2870(75.11)	83(61.03)		85(62.5)	83(61.03)	
Poor	680(17.80)	35(25.74)		30(22.06)	35(25.74)	
**No. of positive LNs**			*0*.*884*			*0*.*474*
0	2324(60.82)	85(62.50)		22(16.18)	22(16.18)	
1–3	907(23.74)	32(23.53)		58(42.65)	61(44.85)	
≥4	590(15.44)	19(13.97)		56(41.18)	53(38.97)	
**Size (cm)**			*0*.*199*			*0*.*924*
2≤2	433(11.33)	22(16.18)		86(63.24)	85(62.5)	
2–5	1887(49.38)	61(44.85)		37(27.21)	32(23.53)	
>5	1501(39.28)	53(38.97)		13(9.56)	19(13.97)	
**Radiotherapy**			*0*.*053*			*0*.*222*
Yes	905(23.68)	42(30.88)		33(24.26)	42(30.88)	
No	2916(76.32)	94(69.12)		103(75.74)	94(69.12)	
**Chemotherapy**			*0*.*011*			*0*.*226*
Yes	853(22.32)	43(31.62)		34(25)	43(31.62)	
No	2968(77.68)	93(68.38)		102(75)	93(68.38)	

SRCC: Signet Ring Cell Carcinoma; No: Number; LN: Lymph Node; RA: Rectal Adenocarcinoma; AA: Anal Adenocarcinoma

Adenocarcinoma was more frequent in patients with RA (90.94% of patients with RA had adenocarcinoma compared with 79.41% of patients with AA), while mucinous adenocarcinoma was more frequent in patients with AA (7.93% of patients with RA had mucinous adenocarcinoma compared with 17.65% of patients with AA, P<0.01). Patients with RA had a lower incidence of either well-differentiated histology (7.09% in RA compared to 13.24% in AA) or poorly differentiated histology (17.80% in RA compared to 25.74% in AA, P<0.01). The use of chemotherapy was significantly greater in patients with AA than in patients with RA (31.62% vs 22.32%, P<0.05). Characteristics of 272 propensity score-matched patients showed no significant differences (Table 1).

**Fig 2:**
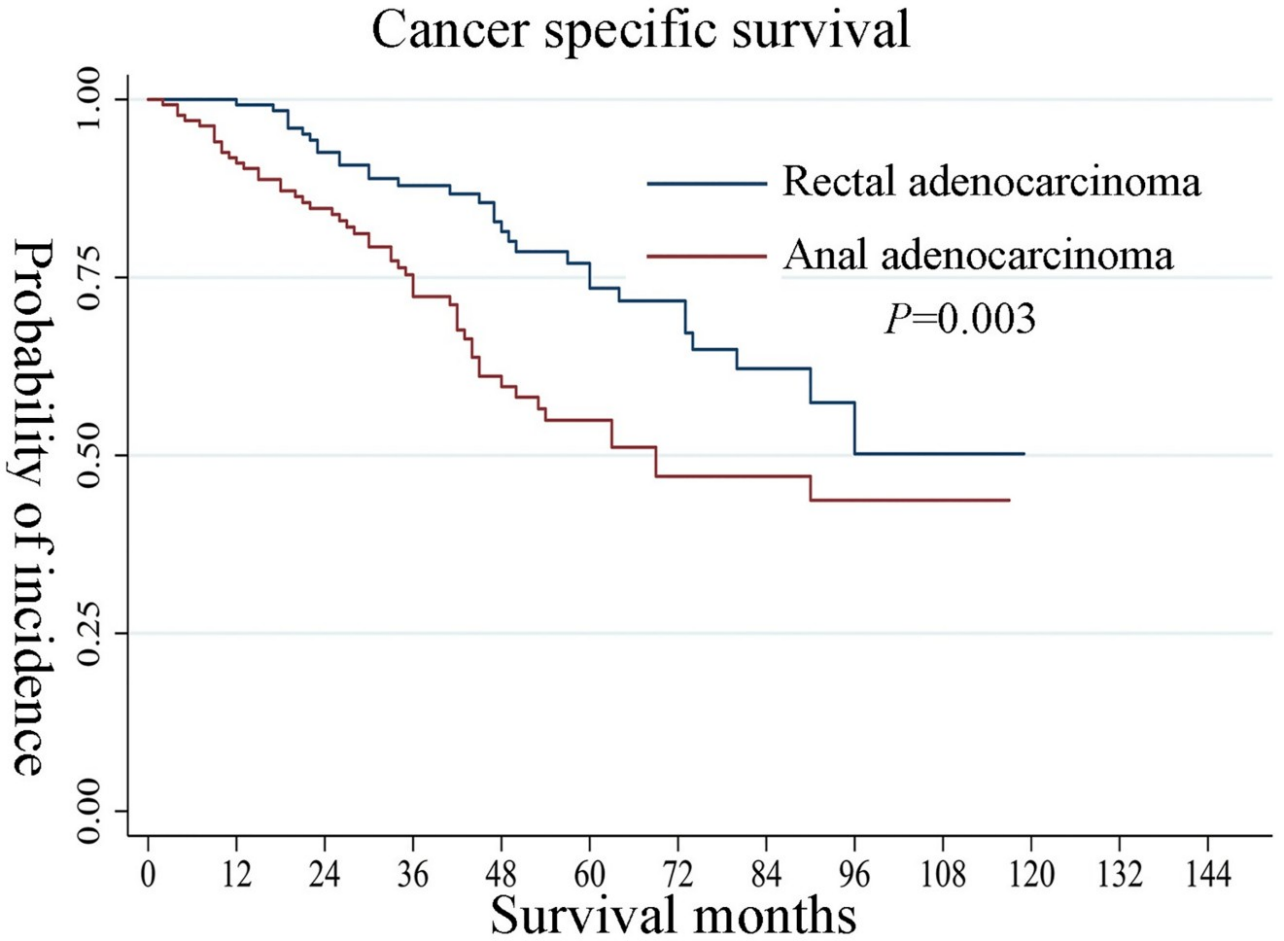
Survival analysis of rectal adenocarcinoma and anal adenocarcinoma.

Table 2 shows the results of the univariate analysis and multivariate Cox regression analyses performed to compare the independent effect of the 2 cancer types (RA and AA) on survival in propensity score-matched patients. Compared with RA, patients with AA had a worse CSS after controlling for age, gender, race, marital status, histology, grade of differentiation, tumor size, number of positive lymph nodes, radiotherapy, and chemotherapy (hazard ratio [HR], 1.96; 95% confidence interval [CI], 1.25–3.07; P<0.01). Increasing tumor size with a cutoff of anal carcinoma (2–5 cm: HR, 0.84; 95% CI, 0.46–1.54; P>0.05; >5 cm: HR, 1.19; 95% CI, 0.66–2.14; P>0.05) had no significant influence on survival. The number of positive lymph nodes (1–3: HR, 2.87; 95% CI, 1.74–4.75; P<0.01; ≥4: HR, 5.50; 95% CI, 3.13–9.67; P<0.01) appeared to significantly influence survival. These results are also presented in Table 3. Fig 2 shows the Kaplan-Meier survival curve among the 2 cancer types, with AA patients having a significantly worse CSS (P value of the log rank test <0.01).

**Table 2:** Univariate and multivariate analyses of predictors of CSS in the study population after propensity score matching (PSM).

Risk Factors	Univariate analysis			Multivariate analysis		
	HR	HR (95% CI)	*P* Value	HR	HR (95% CI)	*P* Value
**Age**						
<50		(-)			(-)	
≥50	1.11	0.64–1.95	*0*.*706*	1.29	0.70–2.36	*0*.*419*
**Gender**						
Female		(-)			(-)	
Male	0.93	0.60–1.44	*0*.*740*	1.43	0.84–2.43	*0*.*192*
**Race**						
White		(-)			(-)	
Black	1.72	0.99–3.01	*0*.*056*	1.39	0.76–2.54	*0*.*286*
Other	1.61	0.84–3.08	*0*.*153*	1.14	0.52–2.51	*0*.*743*
**Marital status**						
Married		(-)			(-)	
Single	1.17	0.60–2.29	*0*.*642*	1.02	0.49–2.11	*0*.*953*
Divorce	1.35	0.85–2.16	*0*.*209*	1.05	0.62–1.79	*0*.*850*
**Histology**						
Adenocarcinoma		(-)			(-)	
Mucinous	1.24	0.73–2.13	*0*.*426*	1.51	0.84–2.74	*0*.*172*
SRCC	2.00	0.73–5.51	*0*.*180*	1.37	0.41–4.62	*0*.*608*
**Grade**						
Well		(-)			(-)	
Moderate	1.34	0.66–2.74	*0*.*422*	1.08	0.51–2.27	*0*.*845*
Poor	2.26	1.06–4.78	*0*.*034*	1.25	0.54–2.91	*0*.*597*
**No. of positive LNs**						
0		(-)			(-)	
1–3	2.87	1.74–4.75	*0*.*000*	3.18	1.78–5.68	*0*.*000*
≥4	5.50	3.13–9.67	*0*.*000*	5.62	2.86–11.05	*0*.*000*
**Size (cm)**						
≤2		(-)			(-)	
2–5	0.84	0.46–1.54	*0*.*563*	0.67	0.35–1.26	*0*.*213*
>5	1.19	0.66–2.14	*0*.*569*	0.79	0.41–1.52	*0*.*483*
**Radiotherapy**						
Yes		(-)			(-)	
No	1.20	0.72–1.98	*0*.*488*	1.71	0.73–4.00	*0*.*213*
**Chemotherapy**						
Yes		(-)			(-)	
No	1.00	0.62–1.61	*0*.*998*	0.58	0.24–1.40	*0*.*228*
**Primary Tumor**						
RA		(-)			(-)	
AA	1.96	1.25–3.07	*0*.*003*	2.06	1.29–3.27	*0*.*002*

SRCC: Signet Ring Cell Carcinoma; No: Number; LN: Lymph Node; RA: Rectal Adenocarcinoma; AA: Anal Adenocarcinoma

**Table 3:** Univariate analysis of predictors of CSS in patients with AA.

Risk Factors	N(%)	Univariate analysis		
		HR	HR (95% CI)	*P* Value
**Age**				
<50	26(19.12)		(-)	
≥50	110(80.88)	1.27	0.62–2.60	*0*.*522*
**Gender**				
Female	51(37.5)		(-)	
Male	85(62.5)	1.07	0.61–1.88	*0*.*807*
**Race**				
White	102(75)		(-)	
Black	20(14.71)	1.47	0.71–3.04	*0*.*299*
Other	14(10.29)	0.83	0.29–2.33	*0*.*720*
**Marital status**				
Married	74(54.41)		(-)	
Single	22(16.18)	0.97	0.42–2.23	*0*.*947*
Divorce	40(29.41)	0.86	0.46–1.59	*0*.*619*
**Histology**				
Adenocarcinoma	108(79.41)		(-)	
Mucinous	24(17.65)	1.08	0.52–2.23	*0*.*838*
SRCC	4(2.94)	1.07	0.26–4.44	*0*.*925*
**Grade**				
Well	18(13.24)		(-)	
Moderate	83(61.03)	1.19	0.46–3.09	*0*.*720*
Poor	35(25.74)	2.18	0.81–5.90	*0*.*123*
**No. of positive LN**				
0	22(16.18)		(-)	
1–3	61(44.85)	2.93	1.55–5.53	*0*.*001*
≥4	53(38.97)	4.24	2.08–8.62	*0*.*000*
**Size (CM)**				
≤2	85(62.5)		(-)	
2–5	32(23.53)	0.62	0.29–1.32	*0*.*211*
>5	19(13.97)	1.01	0.49–2.07	*0*.*985*
**Radiotherapy**				
Yes	42(30.88)		(-)	
No	94(69.12)	1.61	0.84–3.08	*0*.*150*
**Chemotherapy**				
Yes	43(31.62)		(-)	
No	93(68.38)	1.30	0.71–2.39	*0*.*390*

SRCC: Signet Ring Cell Carcinoma; No: Number; LN: Lymph Node; RA: Rectal Adenocarcinoma; AA: Anal Adenocarcinoma

The results of a separate univariate analysis among patients with AA are summarized in Table 3 to illustrate the effect of various patient-related factors on survival. Increasing the tumor size with a cutoff of anal carcinoma (2–5 cm: HR, 0.62; 95% CI, 0.29–1.32; P>0.05; >5 cm: HR, 1.01; 95% CI, 0.49–2.07; P>0.05) had no significant influence on survival. The number of positive lymph nodes (1–3: HR, 2.93; 95% CI, 1.55–5.53; P<0.01; ≥4: HR, 4.24; 95% CI, 2.08–8.62; P<0.01) appeared to significantly influence survival. The use of radiation therapy and chemotherapy among these patients did not appear to significantly influence survival rates (radiotherapy HR, 1.61; 95% CI, 0.84–3.08; P>0.05; chemotherapy HR, 1.30; 95% CI, 0.71–2.39; P>0.05).

## Discussion

This study demonstrated that patients with AA have a worse prognosis than their histological counterparts (patients with RA), even after adjustment for other factors that may affect survival, including age, race, sex, grade, year of diagnosis, and treatment with surgery and/or radiation therapy. The univariate analysis indicated that the T staging criteria for anal carcinoma, which are dominated by tumor size, seem to be invalid for AA, while the number of positive lymph nodes is a factor implying a poor prognosis. The use of radiation therapy and chemotherapy among these patients did not appear to significantly influence survival rates. Our data argue that aggressive therapy should be considered for the treatment of this cancer and that efforts should be made to improve the TNM staging system.

AA, which arises from the anal glands proximal to the anal verge and distal to the dentate line, tends to spread through the submucosa and invade the anorectal wall without an intraluminal mass, which differs significantly from RA. Anal adenocarcinoma occurs predominantly in men compared to anal epidermoid carcinoma, which is commonly found in women and it presents in later stages and has a more aggressive clinical course than epidermoid carcinoma does[3], in addition to which the tendency of progressing rapidly after diagnosis may also be responsible for its poor prognosis. Nonetheless, patients older than 70 years of age have a worse survival[6].

Interestingly, the T staging criteria of anal cancers (mostly squamous cell carcinoma histologically) seem to be invalid for AA, for which the T staging criteria of low-lying RA also have poor applicability on the basis of the different origin, despite the same surgical approach. To the best of our knowledge, there are few studies that discuss the possible factors resulting in poor prognosis for anal adenocarcinoma and note that the existing T staging criteria for anal carcinoma may not be suitable for anal adenocarcinoma. Although anal adenocarcinoma has the same pathology as rectal adenocarcinoma, they differ so much from each other, especially concerning the lymphatic drainage pathway and the N-staging system due to different anatomical locations. The sites of positive lymph nodes dominate the N staging for anal cancers[7]; however, the number of positive lymph nodes also seems to be responsible for the poor prognosis of AA, whose cutoff is referred to that of RA. AA is closest to low-lying RA anatomically and histologically, and the two have a similar surgical approach. They sometimes share the same lymphatic drainage pathway, so low-lying RA was chosen as a control group for a more convincing outcome.

These findings are consistent, to some extent, with those of other studies. The 5-year survival rate of AA in the study was much higher than that reported by Franklin et al[6], which is mainly attributed to radical abdominal perineal resection. However, a strong trend of improved survival was reported among patients with AA who underwent radical surgery, as opposed to local surgical therapies (HR, 0.71; 95% CI, 0.51–1.00; P = 0.05)[6]. Franklin et al[6] also shared their conclusions that AA has a worse prognosis than RA does, and the use of radiation therapy did not significantly influence survival rates.

This study implicates the complication of the N-staging of AA, as the number of positive lymph nodes seems to greatly influence survival, regardless of the fact that the existing N-staging criteria of AA are assessed according to the sites of sites of positive lymph nodes. Therefore, more effort must be made to better understand the relationship. Clinically, cancers in the perianal skin and the anal canal distal to the dentate line, which present with a higher incidence of inguinal node metastasis, drain mainly to the superficial inguinal nodes[8]. The incidence of inguinal node metastases at diagnosis was reported to be 22.6%. The patients with positive inguinal lymph nodes had a poorer survival than that of patients with negative inguinal lymph nodes[2]. The presence of inguinal lymph nodes should be treated with caution, especially in patients with pelvic nodes, regardless of the lymph node size[2]. Several randomized trials have shown that elective irradiation of the groin should be considered for all tumors to reduce inguinal progression risk[9, 10]. Excellent nodal control rates, as high as 98.5% and 100.0%, were achieved in the inguinal region with radiation[11, 12], so elective irradiation of the groin is suggested for local control[13]. Patients with AA were more likely to have distant stage disease and a poorly differentiated malignancy than were patients with RA[6], and there is a lack of a standardized approach to the management of AA, with some who prefer a primarily surgical approach on the basis of the treatment of low-lying rectal cancer and others who base their recommendations on chemoradiation for SCCA. It must be noted that the contemporary National Comprehensive Cancer Network (NCCN) guidelines suggest that the management of anal adenocarcinoma is according to that of the rectal carcinoma so far. Some possible approaches include primary surgery with adjuvant chemoradiotherapy (CRT), primary CRT, and neoadjuvant CRT with surgery and adjuvant CRT[14]. AA has been treated as a low-lying rectal cancer requiring an abdominal perineal resection, and surgery can bring better survival even if only local excision is performed[6]. It seems that radiotherapy and surgery provide the best 5-year disease-free survival rates, reaching 54%[15]. Primary CRT should be combined with APR; otherwise, it may be associated with high rates of local recurrence and distant metastases [1]. Contemporary studies of small sample sizes have suggested chemoradiotherapy mandated before abdominoperineal resection to achieve R0 resections and negative margins, with complete response rates as high as 85% (6/7)[16]. A promising median disease-free survival and median overall survival can be achieved in cases of clear surgical margins[17]. After neoadjuvant therapy, positive circumferential resection margins achieved during abdominoperineal resection were present in only 8% of patients[18]. The current literature suggests that when the disease is potentially curative, radical surgery with either pre- or postsurgery chemoradiotherapy should be attempted to achieve the best overall survival[1]. However, in a retrospective analysis of 22 patients, the conclusions demonstrated that local and regional control with radiation with or without chemotherapy resulted in high relapse rates in AA[3], and it is suggested that the use of radiation therapy among patients with AA did not appear to significantly influence survival rates[6]. Furthermore, patients and doctors pay more attention to the quality of life and have increased concerns about late complications[19, 20]. Therefore, further research is necessary to better understand the role of chemoradiotherapy for AA.

Our study design has several limitations. Due to the strict inclusion criteria, only 136 AA patients were included in this study, which may decrease the degree of power. Retrospective analyses always carry risks of various biases.

The strengths of our study include the following. With the use of a large-scale sample size and PSM method, our study minimized potential biases and had a higher degree of power. Another important merit of the study is the compartmentalization of AA from low-lying RA. The SEER database allowed us to accurately identify patients with adenocarcinoma of the anal canal based on anatomic landmarks and histology, thus avoiding the inclusion of high-lying rectal cancers in our study, which makes the comparison between the two types of cancer more convincing. Nonetheless, all patients included were those who underwent APR, which indicated a radical surgical approach, and potential biases introduced by surgery were minimized by a superselective cohort.

## Conclusion

Based on our analysis of the SEER database, AA confers a significantly worse prognosis than RA does. The T staging criteria of anal carcinoma is dominated by tumor size and seems to be invalid for AA, while the number of positive lymph nodes is a factor implying a poor prognosis.

## Supporting information

S1 File The data use agreement of SEER provided for my data. (PDF)

S2 File The certification of language editing service of my manuscript. (PDF)

S3 File The primary data for analysis I have collected form SEER. (XLSX)
