# Supplementary material for: What factors are associated with the poor prognosis of anal adenocarcinoma compared with low-lying rectal adenocarcinoma based on a population analysis: A propensity score matching study
Source: PLoS One. 2019 Jul 30;14(7):e0219937. doi: 10.1371/journal.pone.0219937 (PMC6667147; doi:10.1371/journal.pone.0219937)
Supplement: S1 File — (PDF) [file pone.0219937.s001.pdf]

Last Name: Wang
SEER ID: 11308-Nov2016
Request Type: Internet Access

SURVEILLANCE, EPIDEMIOLOGY, AND END RESULTS PROGRAM
Data-Use Agreement for the SEER 1973-2014 Research Data File

It is of utmost importance to protect the identities of cancer patients. Every effort has been made to exclude identifying information on individual patients from the computer files. Certain demographic information - such as sex, race, etc. - has been included for research purposes. All research results must be presented or published in a manner that ensures that no individual can be identified. In addition, there must be no attempt either to identify individuals from any computer file or to link with a computer file containing patient identifiers.

In order for the Surveillance, Epidemiology, and End Results Program to provide access to its Research Data File to you, it is necessary that you agree to the following provisions.

1. I will not use - or permit others to use - the data in any way other than for statistical reporting and analysis for research purposes. I must notify the SEER Program if I discover that there has been any other use of the data.
2. I will not present or publish data in which an individual patient can be identified. I will not publish any information on an individual patient, including any information generated on an individual case by the case listing session of SEER*Stat. In addition, I will avoid publication of statistics for very small groups.
3. I will not attempt either to link - or permit others to link - the data with individual level records in another database.
4. I will not attempt to learn the identity of any patient whose cancer data is contained in the supplied file(s).
5. If I inadvertently discover the identity of any patient, then (a) I will make no use of this knowledge, (b) I will notify the SEER Program of the incident, and (c) I will inform no one else of the discovered identity.
6. I will not either release - or permit others to release - the data - in full or in part - to any person except with the written approval of the SEER Program. In particular, all members of a research team who have access to the data must sign this data-use agreement.
7. I will use appropriate safeguards to prevent use or disclosure of the information other than as provided for by this data-use agreement. If accessing the data from a centralized location on a time sharing computer system or LAN with SEER*Stat or another statistical package, I will not share my logon name or password with any other individuals. I will also not allow any other individuals to use my computer account after I have logged on with my logon name and password.
8. For all software provided by the SEER Program, I will not copy it, distribute it, reverse engineer it, profit from its sale or use, or incorporate it in any other software system.
9. I will cite the source of information in all publications. The appropriate citation is associated with the data file used. (Please see either Suggested Citations on the SEER*Stat Help menu or the Readme.txt associated with the ASCII text version of the SEER data.)

My signature indicates that I agree to comply with the above stated provisions.

Qinghua Wang
Signature

2017.5.17
Date

Please print, sign, and date the agreement. Send the form to The SEER Program:

- **By fax to 301-680-9571**
- **Or, e-mail a scanned form to seerfax@imsweb.com**
