# Supplementary material for: What factors are associated with the poor prognosis of anal adenocarcinoma compared with low-lying rectal adenocarcinoma based on a population analysis: A propensity score matching study
Source: PLoS One. 2019 Jul 30;14(7):e0219937. doi: 10.1371/journal.pone.0219937 (PMC6667147; doi:10.1371/journal.pone.0219937)
Supplement: S2 File — (PDF) [file pone.0219937.s002.pdf]

证明

兹证明美国期刊专家（AJE）委托中国国际图书贸易集团有限公司（CIBTC）代开中国税务发票。目前双方的合作方式是：支付宝、银联卡和中国国内银行转账这三种支付方式。

1. 使用支付宝付款的交易，其收款方是：中国国际图书贸易集团有限公司。
2. 使用银联卡付款的交易，其收款方是：瑞购网，该网是中国国际图书贸易集团有限公司旗下网站之一。
3. 使用中国国内银行转账方式的交易，其收款方是：中国国际图书贸易集团有限公司，开户行：中国银行总行营业部，银行账号：778350032951。

不论使用支付宝、银联卡还是银行汇款，税务发票都将由中国国际图书贸易集团有限公司开具。

特此证明!

美国期刊专家（AJE）

2017年5月10日

Certification

American Journal Experts (AJE) hereby authorizes China International Book Trading Cooperation (CIBTC) to issue Chinese tax invoices (Fapiao) on behalf of AJE. The extent of cooperation is currently limited to payments by Alipay, UnionPay and China domestic bank transfer.

Payment recipients of Alipay users are CIBTC. Payment recipients of UnionPay users are Readgo.cn, which is one of the websites that belong to CIBTC. Payment recipients of bank transfer users are: China International Book Trading Cooperation (CIBTC), bank name: Bank of China, account number: 778350032951.

Regardless of payment method, Alipay, UnionPay or bank transfer, all Fapiao will be issued by CIBTC.

Hereby certified,

American Journal experts

May 10, 2017
